# Distinct cortical morphometric inverse divergence changes in Parkinson’s disease correlate with transcriptional expression patterns

**DOI:** 10.1016/j.nicl.2025.103916

**Published:** 2025-11-26

**Authors:** Ting Zou, Xuyang Wang, Xiaofei Hu, Qing Gao, Honghao Han, Huafu Chen, Rong Li

**Affiliations:** a The Clinical Hospital of Chengdu Brain Science Institute, School of Life Science and Technology, University of Electronic Science and Technology of China, Chengdu, PR China; b MOE Key Laboratory for Neuroinformation, Brain-Computer Interface & Brain-Inspired Intelligence Key Laboratory of Sichuan Province, University of Electronic Science and Technology of China, Chengdu, PR China; c Department of Radiology, Southwest Hospital, Third Military Medical University, Chongqing, PR China; d School of Mathematical Sciences, University of Electronic Science and Technology of China, Chengdu, PR China

**Keywords:** Parkinson’s disease, Heterogeneity through discriminant analysis, Morphometric similarity, Partial least squares regression, AHBA dataset

## Abstract

Growing evidence shows that parkinson’s disease (PD) is a heterogeneous neurodegenerative disorder associated with region-specific changes in brain anatomy. However, the genetic mechanisms underlining these abnormalities are unclear. We aim to investigate PD neuroanatomical subtypes and uncover the specific brain-wide gene expression associated with morphometric abnormalities in each PD subtype. The morphometric inverse divergence (MIND) algorithm was used to quantify the morphological similarity based on multiple MRI features in 127 patients with PD and 101 healthy controls (HC). Then, heterogeneity through discriminant analysis (HYDRA) was employed to investigate the PD subtypes based on the MIND strength. Intergroup comparisons were conducted to assess MIND strength and clinical behavioral differences among PD subtypes and HC. Finally, we explored the associations between MIND network changes and gene expression in each PD subtype through partial least squares (PLS) regression, functional enrichment of PLS-weighted genes and transcriptional signature assessment of cell types. We identified two distinct subtypes of PD-related MIND changes, indicating that MIND decreased mainly in the frontal and cingulate cortices in subtype 1, and increased mainly in the occipital cortex and postcentral gyrus in subtype 2 (Bonferroni correction, *p* < 0.05). Both PD subtypes exhibited impaired cognitive function compared to HC, with subtype 2 showing lower Unified Parkinson’s Disease Rating Scale Part III (UPDRS-III) and Hoehn and Yahr (H&Y) scores than subtype 1. Moreover, genetic commonalities analysis were identified 5 shared negative genes in the PD subtypes. Subtype 1 PLS1 genes were functionally enriched in biological processes related to synaptic function, neurodevelopment and degeneration. In addition, subtype 2 PLS1 genes showed additional involvement of metabolic pathways alongside synaptic function. Moreover, we identified MIND-related genes involved in inhibitory and excitatory neurons in subtype 1. In subtype 2, MIND-related genes were involved in astrocytes besides excitatory and inhibitory neurons. Our findings suggest two distinct neuroanatomical subtypes in PD, deepening the understanding of the heterogeneity of PD by bridging the gap between the transcriptome and neuroimaging.

## Highlights

• PD exhibit two distinct MIND-based subtypes.
• Genetic commonalities analysis were identified 5 shared negative genes in PD subtypes.
• Subtype1 PLS1 genes are linked to synaptic function, neurodevelopment/degeneration, and are included in inhibitory/excitatory neurons.
• Subtype2 PLS1 genes are linked to synaptic function and metabolic pathways, and are involved in astrocytes/excitatory/inhibitory neurons.

## Graphical abstract

**Figure:**
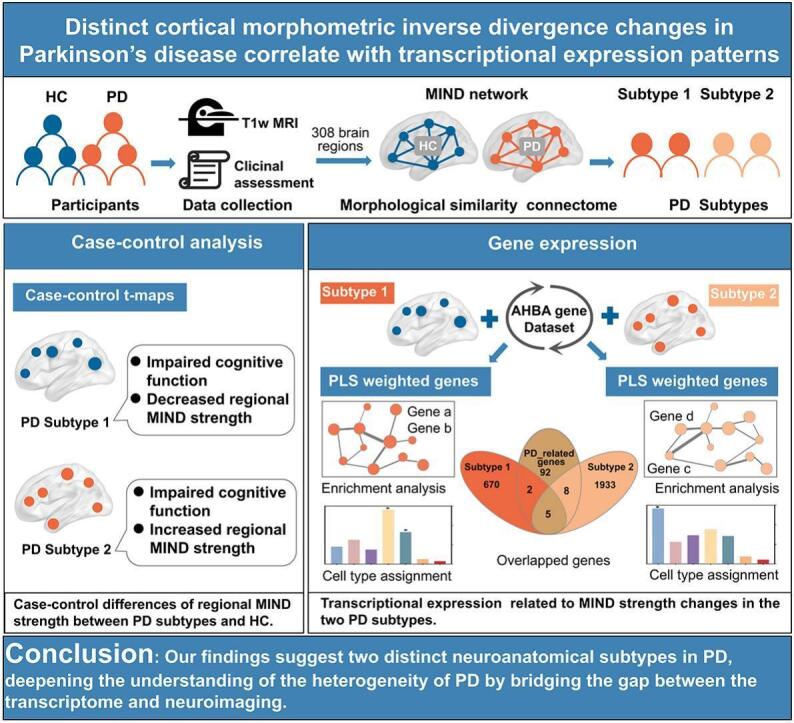

## 1 Introduction

Parkinson’s disease (PD) is a progressive disorder typically characterized by morphological abnormalities in selective brain regions (Oltra et al., 2022, Li et al., 2022). Existing evidence has shown that microscale genetic and cellular mechanisms drive macroscale morphological abnormalities in PD patients (Pisani et al., 2023, Freeze et al., 2019). However, due to high levels of heterogeneity (Yusuke et al., 2024, Su, 2024); the specific vulnerability to brain structural changes and the underlying biological pathogenesis of PD subtypes remain largely unknown.

Structural alterations in gray matter volume, cortical surface area, cortical thickness have been observed in brain regions such as the frontal cortex, parietal lobe, temporal gyrus and occipital cortex in PD (Li et al., 2022, Laansma et al., 2020, Gerrits et al., 2016). However, most studies have only examined single MRI morphometric or anatomical feature at a time, ignoring the associations between multiple MRI parameters. By consolidating multiple MRI anatomical indices measured in each brain region of individuals (Sebenius et al), the previous study conducted MRI-derived similarity network analysis. This analysis showed that MRI similarity can represent architectonic similarity between cortical areas, and that similar areas are more likely to be axonally connected, as predicted by the homophily principle (Sebenius et al). In patients with PD, morphometric similarity network (MSN) analysis revealed decreased morphometric similarity was predominantly in the cingulate, frontal gyrus and temporal regions, increased in parietal and occipital cortical areas (Wang et al., 2024). However, a notable limitation of the MSN methodology stems from its dependence on singular, normalized feature representations per anatomical region. Based on a multidimensional distribution of multiple structural features measured at each vertex, a novel method named morphometric inverse divergence (MIND) was proposed to assess the structural similarity networks, offering enhanced biological interpretability (Sebenius et al., 2023). Building upon prior studies (Sebenius et al., 2023, Yao et al., 2023), the MIND method has the potential to quantify the interregional structural similarity in PD.

Using the multidimensional data including comprehensive clinical evaluations and MRI features, the data-driven modelling identified different PD subtypes with different clinical performances (Shawa et al., 2025, Zhou et al., 2024). Moreover; the heterogeneity through discriminant analysis (HYDRA) characterized pathological heterogeneity by simulating neurobiological deviations from healthy controls (HC) rather than directly clustering of patient populations, a method that has been conventionally applied to other brain disorders (Lalousis et al., 2022, An et al., 2024). However, these studies have only examined one MRI morphometric or anatomical feature at a time. This ignores the potential associations between multiple MRI characteristics that could enhance classification performance. Combining MIND and HYDRA is a promising approach for exploring the PD neuroanatomical subtypes and advancing our understanding of PD pathophysiology.

Genetics and molecular pathology have provided insights into the pathogenesis of PD. Atrophy progression in PD is more obvious in the regions with higher expression of synaptic-related genes, and is negatively correlated with the prevalence of oligodendrocytes and endothelial cells (Tremblay et al., 2021). Furthermore, integrative analyses incorporating MSN and transcriptomic profiling have elucidated putative associations between macroscale neuroanatomical alterations and region-specific gene expression signatures in Parkinson's disease (PD) pathogenesis (Wang et al., 2024). Compared to MSN, the MIND network exhibits a stronger connection to gene co-expression in cortical regions (Sebenius et al., 2023). A combined evaluation of MIND differences and regional gene expression pattern is required to explore potential mechanisms in PD patients.

In our study, we aimed to investigate the potential relationship between the macrostructural alterations and the molecular features in different PD subtypes. First, we constructed individual-level MIND networks to quantify inter-regional cortical similarity patterns across participants. Subsequently, based on the MIND characteristics, we then applied the HYDRA method was to identify distinct PD subtypes and identified the abnormal MIND patterns specific to PD patients compared to healthy controls (HC). For each subtype, partial least squares (PLS) regression was used to explore the specific gene expression patterns spatially related to regional MIND changes in PD. Finally, functional enrichment analysis and specific cell types mapping was performed to characterize how the disease-related genetics drive the morphological phenotype in PD subtypes.

## 2 Materials and methods

### 2.1 Participants

A total of 127 right-handed patients with PD and 101 HC were enrolled in Southwest Hospital of Third Military Medical University. All patients were diagnosed with idiopathic PD by experienced neurologists (according to the Movement Disorder Society criteria in China) (Li et al., 2017). Clinical motor symptoms were evaluated according to the Unified Parkinson's Disease Rating Scale Part III (UPDRS-III) (Fahn, 1987) and Hoehn and Yahr (H&Y) (Hoehn and Yahr, 1967). Cognitive assessment was evaluated using Montreal Cognitive Assessment (MoCA). Levodopa equivalent daily dose (LEDD) for each participant was calculated using an online toolbox (https://www.parkinsonsmeasurement.org/toolBox/levodopaEquivalentDose.htm). The exclusion criteria of our enrolment include: (1) patients with other neurological and psychiatric disorders; (2) patients with obvious complications or adverse events; and (3) patients without full data of demographic characteristics (age and sex, H&Y, UPDRS-III, MoCA and LEDD), preoperative motor evaluations, postoperative motor evaluations, and T1-weighted images. All subjects provided written informed consent before the experiment. The details of demographic characteristics are listed in Table 1.

**Table 1:** Clinical and demographic characteristics of participants.

Variable	HC (n = 101)	subtype 1 (n = 67)	subtype 2 (n = 60)	*p_1_*	*p_2_*	*p_3_*
Age, mean ± SD	58.03 ± 6.03	57.91 ± 8.77	58.92 ± 7.80	0.917^a^	0.143^b^	0.474^b^
Sex (male/female)	44/57	27/40	29/31	0.675^c^	0.557^c^	0.363^c^
MoCA	25.42 ± 1.93	21.22 ± 5.46	21.62 ± 5.17	< 0.001^b^	< 0.001^b^	0.879^b^
Duration	\	7.82 ± 3.66	8.38 ± 3.66	\	\	0.440^b^
Hoehn-Yahr	\	2.60 ± 0.81	2.30 ± 0.73	\	\	0.015^b^
UPDRS III	\	36.91 ± 19.40	29.20 ± 17.20	\	\	0.014^b^
LEDD	\	725.06 ± 233.24	685.43 ± 192.70	\	\	0.377^b^

Abbreviations: PD, Parkinson’s disease; HC, healthy control; UPDRS, United Parkinson’s Disease Rate Scale; Moca, Montreal Cognitive Assessment scale; LEDD, levodopa equivalent daily dose.

*p*_1_ represented the demographics difference between HC and PD subtype 1.

*p*_2_ represented the demographics difference between HC and PD subtype 2.

*p*_3_ represented the demographics difference between PD subtype 1 and PD subtype 2.

a Two sample *t*-test.

b Nonparametric Mann-Whitney tests.

c χ^2^ test.

### 2.2 Imaging acquisition

A 3.0 T Siemens Tim Trio equipment with the magnetization-prepared rapid gradient echo (MPRAGE) sequence in the Southwest Hospital of Third Military Medical University was used to collect T1-weighted structural imaging data. The acquisition parameters were as follows: repetition time (TR) = 1900 ms; echo time (TE) = 2.52 ms; inversion time (TI) = 900 ms; flip angle = 9°; matrix size = 256 × 256; slices = 176; thickness = 1 mm; voxel size = 1 mm × 1 mm × 1 mm.

### 2.3 Imaging preprocessing

**Fig. 1:**
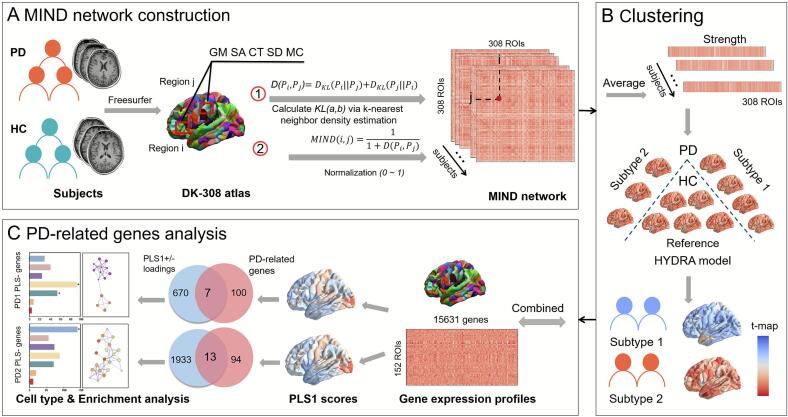
The pipeline for the study. (A) MIND network construction. A 308 × 308 MIND matrix was constructed for each subject with multiple macrostructural features (gray matter volume, surface area, cortical thickness, sulcal depth and mean curvature). (B) Classification. The mean MIND values for each region were calculated by averaging across all connections of the 308 cortical areas. These values were then used as input to the HYDRA model in order to identify PD subtypes. (C) PD-related gene analysis. Partial least squares (PLS) regression was used to identify imaging transcriptomic associations. Functional enrichment of PLS weighted genes and characterization of cell transcription were evaluated to assess the association between brain gene expression and morphometric changes in different PD subtypes.

For each participant, the T1-weighted images were preprocessed in surface-based space using FreeSurfer (v6.0, http://surfer.nmr.mgh.harvard.edu/) through standardized processing pipelines (Fischl, 2012). The cortical preprocessing involved the removal of the skull, the segmentation of tissue, the segmentation of hemibrain and subcortical structures, and the generation of gray-white interfaces and cortical surfaces. Participants were excluded if they had images with poor scan quality. Then, to further check the image quality between groups, the Euler number was calculated for each T1w image (Sánchez et al., 2021, Li et al., 2021). The workflow of our study is shown in Fig. 1.

### 2.4 Construction of MIND network

The cortical surfaces were further divided into 308 spatially adjacent regions by subdividing the 68 cortical regions in the Desikan-Killiany (D-K) atlas (Seidlitz et al., 2018). Then, the parcellated atlas was co-registered into each participant's native space, and the multiple cortical macrostructural indices including gray matter volume, surface area, cortical thickness, sulcal depth and mean curvature were extracted. Subsequently, we normalized the structural features at different scales and estimated the multivariate Kullback-Leibler (KL) divergence between each pair of brain regions. This resulted in a symmetric 308 × 308 MIND matrix for each participant. Finally, the morphometric connectivity strength between pairwise ROIs was normalized to a range of 0 to 1.

### 2.5 Subtyping PD with HYDRA modal

The HYDRA was used to identify the PD subtypes (Drysda et al., 2017, Yao et al., 2023, Varol et al., 2016). For both PD and HC groups, the regional MIND strength was calculated by averaging all the connections between the one region and the other 307 cortical regions, without applying any thresholding in both PD and HC group. Subsequently, the aforementioned features from both the PD and HC group were designated as input features into the modal, where HC and PD participants were assigned respective labels of −1 and 1. The primary parameters were set as follows: 50 iterations between estimating hyperplanes and cluster estimation, 20 clustering consensus steps, 0.25 regularization parameter, and 10-fold cross-validation for performance evaluation. The robustness of the clustering outcomes across the 10-fold cross-validation was evaluated using the Adjusted Rand Index (ARI), which measure the agreement between different clustering results (Chand et al., 2020). In addition, given that age generates non-linear patterns in the brain (Zhou et al., 2023), a quadratic nonlinear model was applied to investigate the developmental trajectories of whole-brain and intra-network MIND strength during the process of ageing.

To validate some of our results, we have randomly dividing the HC and PD patients into two subsets (Subset 1: HC, N = 51, PD, N = 63, Subset 2: HC, N = 50, PD, N = 64) and identified the PD new subtypes by using HYDRA model. Then, we performed spearman correlation analysis between each PD subtype in each Subset.

### 2.6 Case-control analysis of MIND strength for PD subtypes

First, we calculated the average MIND strength in the 308 region and Yeo 7 networks (Schaefer et al., 2018). Then, to examine the group differences between the pooled PD group / PD subtype and HC, we applied the linear regression model (LRM) with averaged MIND strength as the dependent variable and age, gender and TIV as covariates. The model of MIND strength in each region/network (${\text{MIND}}_{\text{i}}$) can be defined mathematically as follows: ${\mathrm{MIND}}_{i}=intercept+\beta 1\times gender+\beta 2\times age+\beta 3\times TIV$. Subsequently, the two sample *t*-tests (contrast = PD − HC) were performed on the adjusted MIND strength at the regional level and network level. Significance was set at *p* < 0.05 with Bonferroni correction for multiple comparisons.

### 2.7 Estimation of regional gene expressions

The brain gene expression data from six postmortem brains at 3702 spatially distinct locations were obtained from the AHBA dataset (http://human.brain-map.org) (Hawrylycz et al., 2012). The Abagen toolbox (https://github.com/rmarkello/abagen) was used to preprocess the AHBA dataset, in accordance with established protocols (Markello et al., 2021). The standardized preprocessing pipeline comprised following steps: (i) converting the microarray probes into gene symbols, (ii) excluding low-intensity probes with expression below the background noise in over 50 % of samples, (iii) selecting probes with the highest homogeneities of regional variation for genes targeted by multiple probes, (iv) assigning the samples to brain regions within 2 mm Euclidean distance from the region boundary (Tamnes and Agartz, 2016), (v) normalizing gene expression across tissue samples using a scaled robust sigmoid function. Given the limited availability of right hemispheric samples in the AHBA dataset (Arnatkeviciute et al., 2019), our analysis was restricted to the left hemisphere. Therefore, a transcriptional matrix consisting of 152 left brain regions and 15,631 gene expression profiles was obtained.

### 2.8 The relationship between MIND strength and transcriptome

PLS regression (Abdi and Williams, 2013) was applied to investigate the relationship between regional MIND changes (response variables) and expression of all 15,631 genes (predictor variables) genes. To evaluate the variability of each gene in the first PLS component (PLS1), bootstrapping (5000 times) was employed (Li et al., 2025). Then, we calculated the ratio of the PLS1 weight of each gene to its bootstrap standard error, and ranked all genes based on their contribution to PLS1 (Morgan et al., 2019). The spatial correlation analysis between each MIND *t*-statistic maps and PLS scores was performed by Pearson’s correlation method. In addition, according to the PLS1 weight scores, the significant genes were selected as PLS1+ genes (Z > 3, *p*_FDR_ < 0.005) and PLS1- genes (Z <  -3, *p*_FDR_ < 0.005).

By searching “Parkinson disease” under “Gene Classifications” in the AHBA dataset (https://human.brain-map.org/microarray/search), a total of 107 unique PD-related genes were obtained. Hence, the overlapped genes were selected as the intersection between these 107 PD-related genes and the significant PLS1 genes. Pearson's correlation analysis was then applied to estimate the relationship between the *t*-statistic map for each PD subtype and its corresponding overlapping gene expression. To identify whether the altered MIND-related gene expression is similar in the pooled PD group and in PD subtypes, we also applied PLS regression to regional MIND changes in the pooled PD group. Statistical significance was set at *p* < 0.05, with FDR correction for multiple comparisons.

### 2.9 Functional enrichment evaluation of PLS1 positive or negative genes

The functional enrichment analysis was conducted using the automated *meta*-analysis tool Metascape (https://metascape.org/) (Zhou et al., 2019); with the PLS1 genes (Z > 3 or Z < -3, *p*_FDR_ < 0.005) as the input. The selected databases encompassed the Gene ontology (GO) for biological processes and the Kyoto Encyclopedia of Genes and Genomes (KEGG). The significance threshold for the obtained enrichment pathways was set at 5 %, adjusted by FDR correction.

### 2.10 Assigning PD-related genes to cell types

Following the procedure in (Wang et al., 2024); cells were classified into seven types, namely astrocytes, endothelial cells, microglia, excitatory neurons, inhibitory neurons, oligodendrocytes, and oligodendrocyte precursors (OPCs). To identify the above cell types to which these genes could be attributed in each PD subtype / the pooled PD group, we overlapped the PLS1- or PLS1+ ranked gene lists with the gene sets of the above cell types. To assess the significance of overlapping genes across cell types, *p*-values were obtained using permutation testing, followed by false discovery rate (FDR) correction (adjusted *p* < 0.05, 5000 rotations). Similarly, the enrichment analysis of overlapping genes was conducted using Metascape (https://metascape.org). Statistically significant GO terms and KEGG pathways for each cellular population were determined, with a FDR correction threshold of *p* < 0.05 applied.

## 3 Results

### 3.1 Demographic and clinical data analysis

**Fig. 2:**
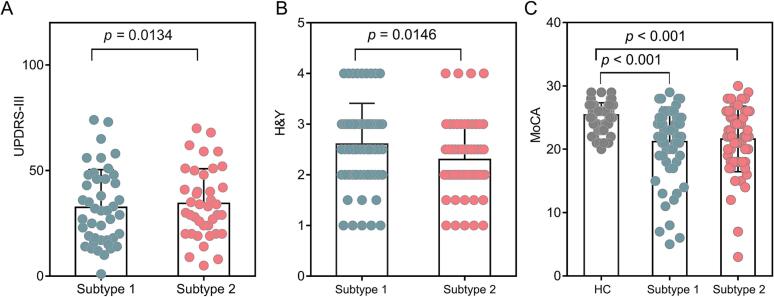
Comparison of clinical scales for between PD subtypes and HC. A-B. Compared to subtype 1, subtype 2 exhibited decreased Unified Parkinson's Disease Rating Scale (UPDRS-III) and Hoehn and Yahr (H&Y) stages. C. Compared to HC, the plot shows decrease of Montreal Cognitive Assessment (MoCA) scores in both PD subtypes.

The adjusted rand index (ARI) was used to determine the optimal cluster (2–10 clusters). The highest ARI value (0.754) was obtained for a value of K set at 2 (Supplementary Fig. S1). Consequently, 67 PD patients were assigned to subtype 1, and 60 to subtype 2. For the statistical significance, significant MIND changes were examined by the two-sample *t*-test or nonparametric Mann-Whitney tests, depending on whether the data were normally distributed (Emerson, 2016). The demographic analysis showed no differ significantly in age (*t* = 0.104; *p_1_* = 0.917; *u* = 1.46, *p_2_* = 0.143), sex (*χ^2^* = 0.176, *p_1_* = 0.675; *χ^2^* = 0.345, *p_2_* = 0.557) between each PD subtypes and HC. We found no significant differences in the Euler number between the pooled PD group / PD subtype 1/ PD subtype 2 and HC (Supplementary Fig. S2). Both PD subtypes exhibited significantly decreased MoCA scores compared with HC. In addition, subtype 1 and subtype 2 exhibited no significant difference in sex, age, duration, MoCA and LEDD scores. The subtype 2 displayed lower H&Y score and UPDRS-III score than subtype 1 (Fig. 2).

### 3.2 Subtyping PD-related changes in MIND strength

**Fig. 3:**
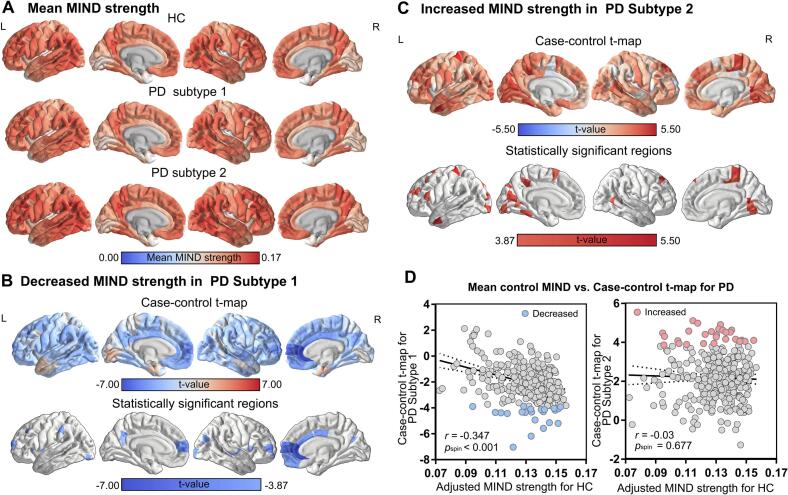
Case-control differences of regional MIND strength. (A) The cortical map of the mean MIND values in controls and PD subtypes. (B-C) Case-control comparison (t-map) of regional MIND for subtype 1 and subtype 2 respectively. (D) The mean control regional MIND exhibited a negative correlation with the t-statistics map in subtype 1, yet no correlation with the t-statistics map in subtype 2. HC, Healthy control; PD, Parkinson’s disease.

The group average MIND over 308 ROIs is shown in Fig. 3A. The regional MIND distributions in patients with PD demonstrated resemblance to those observed in HC, exhibiting a range from 0 to 0.17 in both HC and PD patients within the present study. In all groups, MIND varied across cortical areas, with a higher score in frontal lobe and parietal lobe and lower score in temporal pole and insula. The quadratic non-linear model showed only the global strength of HC exhibited decreasing trend during ageing. Moreover, except visual network, the MIND strength in other network of HC displayed a pronounced and consistent decline. The limbic network of subtype 2 exhibited a rapid increase, subsequently followed by a progressive decline (Supplementary Fig. S3). Compared with HC, the global MIND strength of subtype 1 was reduced, whereas that of subtype 2 was increased (Supplementary Fig. S4). Furthermore, significantly decreased MIND strength in subtype 1 was observed in the left rostral middle frontal cortex, superior frontal cortex and right inferior parietal cortex, medial orbitofrontal cortex, posterior cingulate cortex, rostral anterior cingulate cortex and superior frontal cortex (Fig. 3B and Supplementary Table S1, Bonferroni correction, *p* < 0.05). Conversely, patients with subtype 2 exhibited significantly increased regional MIND strength in the left lateral occipital cortex, lingual gyrus, postcentral gyrus, rostral middle frontal cortex, superior parietal cortex, and the right fusiform, lingual gyrus, paracentral cortex and superior frontal cortex (Fig. 3C and Supplementary Table S1, Bonferroni correction, *p* < 0.05). Interestingly, the MIND strength of HC and the case-control *t*-map of subtype 1 exhibited a negative and spatial correlation (*r* = -0.347, *p_spin_* < 0.001), whereas there was no correlation between the MIND strength of HC and case-control *t*-map of subtype 2 (Fig. 3D).

Compared to HC, the subtype 1 subtype displayed significantly decreased MIND strength in functional Yeo 7 networks (Schaefer et al., 2018); while subtype 2 displayed increased MIND strength (Supplementary Fig. S5, Bonferroni correction, *p* < 0.05). Additionally, we explored the spatial correlation between the correlation coefficient maps of MIND strength and the motor/non-motor scores and case-control *t*-maps of PD subtypes. In subtype 1 patients, the case-control *t*-map displayed a significant negative correlation with the correlation maps between MIND strength and UPDRS-III/H&Y scores, and a positive correlation with the correlation maps between MIND strength and MoCA scores(Supplementary Fig. S6A). In contrast, no such relationship was identified in subtype 2 patients (Supplementary Fig. S6B).

The validation analysis also revealed that K = 2 exhibited the highest reproducibility of PD (Supplementary Fig. S7). Consequently, 29 PD patients were assigned to subtype 1 and 34 PD patients to subtype 2 in Subset 1, and 34 PD patients were assigned to subtype 1 and 30 PD patients to subtype 2 in Subset 2. The verification analysis showed that the distribution of regional MIND changes in each corresponding PD subtype was similar to the main research results. The case-control t-map for PD subtype 1 displayed positive spatial correlations with that of subtype 1 in Subset 1 (*r* = 0.741, *p_spin_* < 0.001) and subtype 1 in Subset 2 (*r* = 0.734, *p_spin_* < 0.001). The case-control t-map for PD subtype 2 displayed positive spatial correlations with that of subtype 2 in Subset 1 (*r* = 0.654, *p_spin_* < 0.001) and subtype 2 in Subset 2 (*r* = 0.749, *p_spin_* < 0.001) (Supplementary Fig. S8).

### 3.3 Transcriptional patterns related to regional changes in MIND strength

**Fig. 4:**
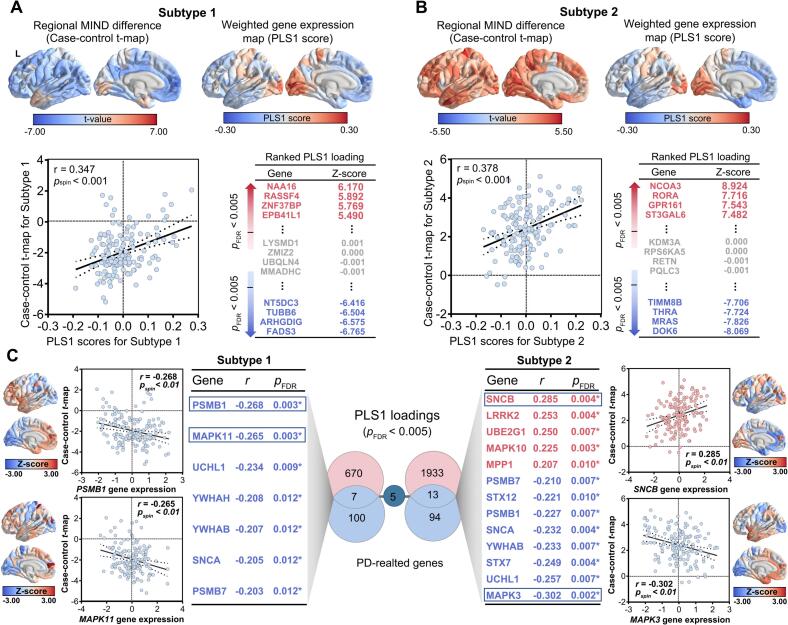
Transcriptional expression patterns related to differences in MIND strength. (A-B) Cortical case-control t-maps and regional PLS1 gene expression weighted values in the left hemisphere for the subtype 1 and subtype 2. Scatterplots showing the significant spatial correlation between PLS1 scores and the case-control t-maps of MIND strength in both PD subtypes. The PLS1 gene expression weights in subtype 1 and subtype 2 subtypes. (C) Spearman’s correlation analysis showed the expression of PD-related genes from AHBA datasets was positively or negatively associated with regional changes in MIND. Subtype 1 showed 7 negative genes and subtype 2 showed 5 positive genes and 8 negative genes. The subtype 1 and subtype 2 subtypes exhibited 5 overlapped negative genes. All p-values were obtained from spatial correlation tests and adjusted with FDR correction. PD, Parkinson’s disease. * indicates *p* < 0.05.

Using case-control *t*-maps in MIND strength for PD patients, a PLS regression analysis was conducted to identify the PD-related gene. In our multivariate analysis, only PLS1 reached statistical significance. Moreover, the PLS1 was identified as a linear combination of gene expression exhibiting the strongest association with *t*-statistic maps in PD (permutation test, 5000 rotations, *p* < 0.05). The PLS1 of two subtypes respectively explained 13 % and 14 % of the variations in the MIND strength changes, and PLS1 of the pooled PD group effectively explained 15 %. The regional PLS1 weighted map in PD subtypes showed an anterior-posterior gradient of gene expression (Fig. 4A-B). Irrespective of PD subtype 1 or PD subtype 2 patients, the PLS1 scores exhibited a significant spatial correlation with the case-control *t*-value maps in MIND strength (subtype 1, Spearman’s *r* = 0.362, *p_spin_* < 0.001, subtype 2, Spearman’s *r* = 0.378, *p_spin_* < 0.001; Fig. 4C, Supplementary Table S2). Then, we identified a 230 PLS1+ gene set (Z > 3) and a 447 PLS1-  gene set (Z <  -3) in subtype 1, and a 913 PLS1+ gene set, and a 1033 PLS1 − gene set in subtype 2 after normalizing the PLS1 weights (all *p_FDR_* < 0.005; Fig. 4D, and Supplementary Table S3). In addition, the gene analysis result of the pooled PD group was presented in Supplementary Fig. S9. An intersection analysis of these results identified a common set of genes associated with MIND strength differences across PD subtype 1, PD subtype 2, and the overall PD cohort, comprising 114 PLS1 + genes and 259 PLS1- genes (Supplementary Fig. S10).

Subsequently, 7 overlapping genes from the known 107 PD-related genes and 677 PLS1 genes was identified in subtype 1. Of these, expression in seven genes (PSMB1, MAPK11, UCHL1, YWHAH, YWHAB, SNCA, PSMB7) showed negative correlations with MIND changes in subtype 1. In subtype 2, A total of 13 overlapping genes were identified between the 1946 PLS1 genes and the 107 known PD-related genes, including 5 positive genes (SNCB, LRRK2, UBE2G1, MAPK10, MPP1) and 8 negative genes (PSMB7, STX12, PSMB1, SNCA, YWHAB, STX7, UCHL1, MAPK3) (all *p_FDR_* < 0.05; Fig. 4E). Moreover, subtype 1 and subtype 2 displayed 5 shared negative genes (PSMB7, PSMB1, SNCA, YWHAB, UCHL1). We only showed the top 2 positively (or negatively) correlations between the expression of overlapping genes and the regional changes in MIND strength, respectively.

### 3.4 Functional enrichment of genes correlated with regional changes in MIND strength

**Fig. 5:**
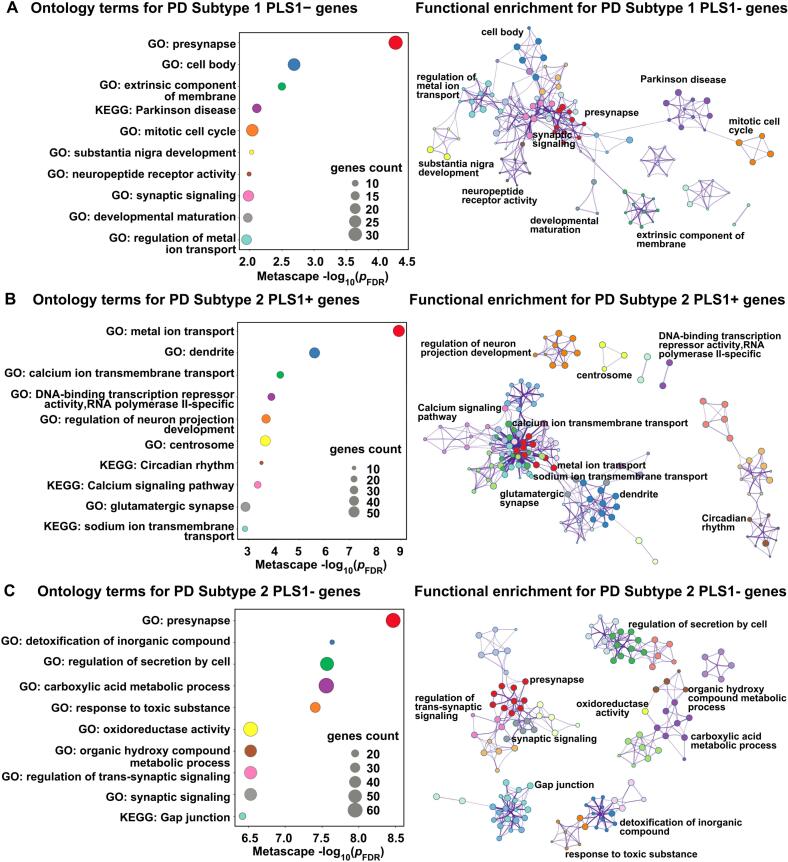
Functional enrichment of PLS1 weighted genes related to regional changes in MIND. The top 10 ontology terms for PLS1- genes (Z < -3, *p_FDR_* < 0.005) / PLS1+ genes (Z > 3, *p_FDR_* < 0.005) in PD subtype1 (A) and PD subtype2 (B-C). Metascape enrichment network visualization showing the same color belong to the same cluster. The size of the circle represents the number of genes involved in a given term.

For subtype 1 patients, only functional enrichment of PLS1- genes reached statistically significant. We showed the top 10 significant terms, and the PLS1- genes were mainly enriched in the GO biological processes (BP), such as “presynapse”, “substantia nigra development”, “synaptic signaling”, “developmental maturation”, “regulation of metal ion transport”, and significantly enriched in one KEGG pathway, which was “Parkinson’s disease” (*p* < 0.05, Fig. 5A). In subtype 2 cohorts, the enrichment analysis revealed that BP terms (e.g., “metal ion transport”, “calcium ion transmembrane transport”, “DNA-binding transcription repressor activity, RNA polymerase II-specific”, “regulation of neuron projection development”, “glutamatergic synapse”) and KEGG pathway (e.g., “calcium signaling pathway”) for PLS1+ gene lists (*p* < 0.05, Fig. 5B). Moreover, the subtype 2 PLS1- genes were significantly enriched in synapse −related functions, such as “presynapse”, “regulation of *trans*-synaptic signaling”, “regulation of *trans*-synaptic signaling”, “synaptic signaling”, “gap junction” and others (*p* < 0.05, Fig. 5C). In addition, functional enrichment of PLS1 weighted genes related to regional MIND changes of the pooled PD group was shown in Supplementary Fig. S11.

### 3.5 Cell-type-specificity of genes related to regional changes in MIND strength

**Fig. 6:**
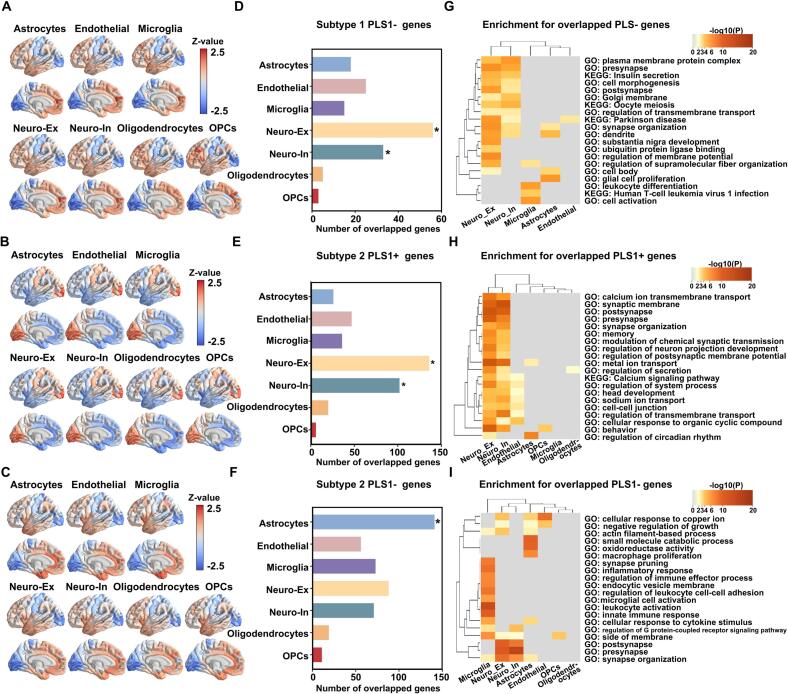
Cell type-specific expression associated with MIND changes. (A-C) Cortical maps showing regional gene expression for each cell type by overlapping PLS1 genes and genes specific to each cell type. (D) The number of overlapping genes with PLS1- weighted genes for each cell type in subtype 1, including astrocytes (number = 18, adjusted *p*_perm_ = 1); endothelial cells (number = 25, adjusted *p*_perm_ = 0.946, 5000 rotations); microglia (number = 15, adjusted *p*_perm_ = 1); excitatory neurons (number = 56, adjusted *p*_perm_ < 0.001); inhibitory neurons (number = 33, adjusted *p*_perm_ = 0.002); oligodendrocytes (number = 5, adjusted *p*_perm_ = 1); OPCs (number = 3, adjusted *p*_perm_ = 1). (E) The number of overlapping genes with PLS+ weighted genes for each cell type in subtype 2, including astrocytes (number = 26, adjusted *p*_perm_ = 1);endothelial cells (number = 47, adjusted *p*_perm_ = 1); microglia (number = 36, adjusted *p*_perm_ = 1); excitatory neurons (number = 136, adjusted *p*_perm_ < 0.001); inhibitory neurons (number = 102, adjusted *p*_perm_ = < 0.001); oligodendrocytes (number = 20, adjusted *p*_perm_ = 1); OPCs (number = 6, adjusted *p*_perm_ = 1). (F) The number of overlapping genes with PLS- weighted genes for each cell type in subtype 2, including astrocytes (number = 141, adjusted *p*_perm_ < 0.001);endothelial cells (number = 56, adjusted *p*_perm_ = 1); microglia (number = 73, adjusted *p*_perm_ = 0.237); excitatory neurons (number = 88, adjusted *p*_perm_ = 0.186); inhibitory neurons (number = 71, adjusted *p*_perm_ = 0.178); oligodendrocytes (number = 19, adjusted *p*_perm_ = 1); OPCs (number = 11, adjusted *p*_perm_ = 0.731). (G-I) Gene ontology and pathway terms enriched for changes in MIND-related genes for the different cell types. All *p*-values were obtained from permutation tests and adjusted with FDR correction (*p* < 0.05).

The present study assigned PLS1 genes to seven canonical cell classes, thereby obtaining regional gene expression maps for each cell type from overlapping genes between PLS1+/PLS1- genes and each cell type-specific gene (Fig. 6A-C). For PD subtype 1, 56 PLS1- genes were significantly involved in excitatory neurons (Fig. 6D, *p*_perm_ < 0.001, FDR-corrected) and 33 genes were significantly involved in inhibitory neurons (Fig. 6D, *p*_perm_ = 0.002, FDR-corrected). Subsequently, the cell-specific gene enrichment analysis revealed that MIND strength alterations in subtype 1 were significantly enriched in BPs associated with “presynapse”, “postsynapse”, “dendrite”, “substantia nigra development”, and significantly enriched in one KEGG pathway, which was “Parkinson’s disease” (Fig. 6G). For subtype 2 patients, 136 PLS1+ genes were significantly involved in excitatory neurons (Fig. 6E, *p*_perm_ < 0.001, FDR-corrected) and 102 genes were significantly involved in inhibitory neurons (Fig. 6E, *p*_perm_ < 0.001, FDR-corrected). Enrichment analysis enriched in BPs including “synaptic membrane”, “postsynapse”, “presynapse”, “synapse organization” and “modulation of chemical synaptic transmission” (Fig. 6H). In the PLS1- genes list, 141 genes were significantly assigned to astrocytes (Fig. 6F, *p*_perm_ < 0.001, FDR-corrected). Astrocyte-specific genes were enriched in “inflammatory response”, “leukocyte activation”, “innate immune response” and others (Fig. 6I). Moreover, cell type-specific expression associated with MIND changes of the pooled PD group was shown in Supplementary Fig. S12.

## 4 Discussion

In our study, we investigated the potential relationships between regional MIND changes and the corresponding transcriptomic patterns for each PD subtype. We applied HYDRA model to cluster PD patients into two distinct subtypes, showing contrasting abnormal MIND patterns compared with HC individuals. Moreover, the AHBA dataset was utilized to identify PD-related genes for each subtype, thereby facilitating the determination of the genetic commonalities and specificities between subtype 1 and subtype 2 subtypes. Subtype 1-related genes were primarily enriched in pathways associated with synaptic function, neurodevelopment and degeneration. Furthermore, these genes were mapped to the distribution of neuronal cells. Subtype 2-related genes were primarily enriched in synaptic function and metabolism-related pathways and were mapped to the distribution of astrocytes and neuronal cells. Moreover, the two subtypes shared common pathophysiological foundations with the pooled PD group, but also exhibit distinct molecular signatures. The shared enrichment for synaptic function and neuronal cell types likely reflects a core, common pathophysiology underlying PD, and the different enrichments remain new findings, as they provide a potential biological rationale for the clinically and morphometrically distinct subtypes we identified. These findings reveal MIND changes in different PD subtypes, shedding light on the genetic homogeneity and heterogeneity of PD.

Our analysis identified two distinct PD subtypes, and the developmental trajectories analysis revealed that the MIND strength in HC was a consistent downward trajectory across the observed age spectrum, but not in PD subtypes. This finding suggests that there are disease-specific deviations from normal ageing trajectories, whereby the brain structure demonstrates progressive decline with advancing age (Bethlehem et al., 2022). The MIND intensity differs diametrically between the two PD subtypes and HC at both the regional and network levels. In our study, we also found that both of the two PD subtypes showed a decrease in MoCA scores when compared with HC. However, subtype 2 showed reduced UPDRS-III scores yet equivalent MoCA scores in comparison to the subtype 1 group. The case-control *t*-map displayed a significant negative correlation with the correlation maps between MIND strength and UPDRS-III/H&Y only in subtype 1, suggesting that motor characteristics distinguished the two specific PD subtypes. Specifically, significantly decreased MIND strength in subtype 1 was mainly observed in the frontal cortex and cingulate cortex, suggesting that these cortical areas are less interconnected or more differentiated from other brain regions. The observed alterations in the patterns of similarity within these brain regions are consistent with the results measured by MSN (Wang et al., 2024). Previous study indicates that cortical regions with comparable morphometric properties tend to share cytoarchitectonic classifications and exhibit stronger anatomical connectivity (Wei et al., 2018). Decreased similarity strength may indicated increased architectonic specialization and diminished axonal connectivity with other cortical regions (Lin et al., 2024). Moreover, the DTI study also corroborated the hypothesis that PD involved weakened structural connectivity in frontal and cingulate network, which is associated with cognitive decline (Agosta et al., 2014, Tessitore et al., 2016). Subtype 2 exhibited an atypical and irregular increases in MIND strength, mainly in the occipital cortex and postcentral gyrus. This may imply heightened cytoarchitectural similarity and axial connectivity between these brain structures, reflecting a compensatory mechanism that prevents behavioral changes (Jastrzbowska et al., 2019).

The analysis of PD-related genes revealed specific transcriptional patterns in PD subtypes. Regional MIND changes were associated with PD-related genes (7/107 in subtype 1 and 13/107 in subtype 2), including a shared subset genes (5/107). The expression of the 5 genes exhibited negative associations with corresponding changes in the MIND strength among different PD subtypes, suggesting that dysregulation of these shared PD-related pathogenic genes is associated with macrostructural brain abnormalities in the different PD forms subtypes. Pathologically, the SNCA gene, which encoded α-synuclein, is relatively common in PD and has been linked to the severity of PD (Dehay et al., 2015). SNCA mutations alter α-synuclein expression, structure, and properties, generating toxic aggregates that impair dopaminergic neuron function in PD (Appel-Cresswell et al., 2013). The UCHL1 gene has been demonstrated to be associated with neurodegeneration, and a recent metabolic analysis indicated that UCHL1 functions as an integrative factor, connecting glycolysis and PD pathology (Ham et al., 2021). Moreover, the PSMB1 in the PD transcriptomic datasets (GSE54536) can significantly predict the occurrence of PD (Yuan et al., 2020). In addition to the genetic commonalities, we identified specific gene expression in the two PD subtypes. The expression of YWHAH was specifically associated with MIND changes in subtype 1. It is reported that YWHAH is a candidate biomarker for early disease detection and a therapeutic target worthy of further mechanistic characterization in neurodevelopmental and neurodegenerative disorders (Nguyen et al., 2023). Only positive genes was found in subtype 2. By restoring auxilin function in mutant LRRK2 dopaminergic neurons, the athogenic phenotypes in PD were partially alleviated (Nguyen and Krainc, 2018). In addition, the reduction of LRRK2 activity or expression is neuroprotective (Tolosa et al., 2020), and may play a role in maintaining structural similarity. Our findings provide further evidence to suggest that region-specific gene expression patterns could drive PD subtypes through selective neuronal susceptibility.

PLS1 weighted gene analysis revealed enriched biological processes and KEGG pathways that help to interpret transcriptional signatures associated with MIND strength variations across PD subtypes. The PLS1- weighted genes in subtype 1 and subtype 2 were enriched in overlapping ontological terms, including “presynapse” and “synaptic signaling”. Previous studies have demonstrated that α-Synuclein accumulation in presynaptic terminals is associated with disrupted synaptic protein function, altered dopamine neurotransmission and degeneration of striatal dopaminergic neurons (Alabi and Tsien, 2012), which leads to subsequent dysfunction in PD (Bloem et al., 2021, Wang et al., 2025). By interfering with synaptic vesicle recycling and release, α-synuclein disrupts presynaptic signaling (Murphy et al., 2000). In addition, the subtype 1 functionally enriched in nervous system development related biological process and one KEGG pathway (“Parkinson disease”), suggesting that subtype 1 could be a typical subtype of PD. The abnormal development or degeneration of dopaminergic neurons in the substantia nigra pars compacta (SNc) forms the basis of the pathology of PD and contributes to Parkinson's disease-like motor dysfunction (Suda et al., 2018). Only PLS1+ weighted genes in subtype 2 were enriched in the GO biological processes associated with calcium signaling dysregulation and abnormal transcriptional regulation. A pathological study revealed that targeting calcium dyshomeostasis that arising from dysfunctional organelles or miscommunication between organelles has the potential to restore proper calcium homeostasis and improve neuronal function (Kaur et al., 2023).

Cellular abnormalities have been known to play a pivotal role in the PD development. Excitatory and inhibitory neuron constituted the majority in both PLS1- weighted genes in subtype 1 and PLS1+ weighted genes in subtype 2. Existing evidence indicates that excitatory/ inhibitory (E/I) balance effectively sustains homeostasis in the central nervous system, and the E/I imbalance of various neurotransmitters, such as dopamine, glutamate, GABA, acetylcholine and serotonin, is related to the pathological progression of PD (Yizhar et al., 2011, Murueta-Goyena et al., 2019). Our result suggest the differential mechanisms of compensatory adaptation of neural networks in different subtypes of PD. Moreover, the PLS1- genes were significantly involved in astrocytes in subtype 2. Consistent with previous study, which revealed that genes known to show a causative role in the development of PD are expressed in astrocytes (Booth et al., 2017). In addition to modulating Ca^2+^ and K^+^ homeostasis (Chan et al., 2009), astrocytes can act as a barrier to prevent toxic signals from spreading to the surrounding healthy tissues when microglia trigger an immune response (Sofroniew and Vinters, 2010). The both subtypes displayed similar profiles of enrichment (i.e., synaptic function, excitatory/inhibitory neurons), suggesting their findings largely reflect a general PD biology with a secondary enrichment for astrocytes/metabolic function. In addition, the distinct enrichments (e.g., in metabolic pathways and astrocytes) may provide a potential biological rationale for the clinically and morphometrically distinct subtypes that we identified. These findings provided us with an accountable pattern to investigate cellular transcriptional profiles in PD patients.

Some limitations should be noted in current study. First, although it was sufficient to display significant reductions in the PD subtype group, the sample size for classification using machine learning methods is still relatively small. Second, we used public available genetic data of the left brain from the AHBA dataset, which was obtained from the post-mortem brains of six donors with no history of neurological disease. Thus, the relationship between genes and MIND-related changes may not represent the brain condition. Further in vivo and individual PD gene investigation are essential to validate our findings. In addition, neuroimaging studies have reported that PD undergoes structural changes in subcortical brain regions (Hm et al., 2014, Lee et al., 2011). While the MIND method effectively captures cortical morphology, its cortical feature basis prevents the investigation of subcortical regions. Future developments may create unified models for whole-brain morphological similarity analysis. Finally, our study was also based on patients with non-first-episode PD. Although there was no significant difference in the intake of dopaminergic medications between the two subtypes, follow-up studies are needed to clarify the association between MIND changes and drug influence.

## 5 Conclusion

In summary, two distinct subtypes of MIND changes were identified, indicating that MIND was mainly decreased in the frontal cortex and cingulate cortex in subtype 1 and increased in the occipital cortex and postcentral gyrus. When combining the morphometric information with transcriptional signatures, the gene enrichment profiles revealed that were characterized by a combination of overarching pathways common to PD and subtype-specific biological processes. We discovered that MIND-related genes were functionally enriched in biological processes related to synaptic function, neurodevelopment and degeneration, and involved in inhibitory and excitatory neurons in subtype 1. For the subtype 2, MIND-related genes were functionally enriched in biological processes related to synaptic function and metabolism-related pathways, and were involved in astrocytes as well as excitatory and inhibitory neurons. These findings revealed MIND changes in different PD subtypes, providing valuable insights to advance our understanding of PD heterogeneity.

## 6 Patient consent statement

All the subjects provided written informed consent before the experiment.

## Ethics approval statement

Our study was reviewed and approved by the institutional ethics committee of the Southwest Hospital of Third Military Medical University.

## Funding statement

This work was supported by the National Natural Science Foundation of China (Nos. 82372085, 62333003, 62036003, 62173070).

## CRediT authorship contribution statement

**Ting Zou:** Writing – review & editing, Writing – original draft, Visualization, Validation, Methodology, Investigation, Formal analysis, Conceptualization. **Xuyang Wang:** Writing – review & editing, Visualization, Validation, Methodology, Formal analysis. **Xiaofei Hu:** Writing – review & editing, Investigation, Data curation, Conceptualization. **Qing Gao:** Writing – review & editing, Methodology, Funding acquisition. **Honghao Han:** Writing – review & editing, Visualization, Validation, Formal analysis. **Huafu Chen:** Writing – review & editing, Supervision, Funding acquisition. **Rong Li:** Writing – review & editing, Supervision, Funding acquisition, Conceptualization.

## Declaration of competing interest

The authors declare that they have no known competing financial interests or personal relationships that could have appeared to influence the work reported in this paper.

## Data Availability

The data that support the findings of this study are available from the corresponding author upon reasonable request.
