# Supplementary material for: Distinct cortical morphometric inverse divergence changes in Parkinson’s disease correlate with transcriptional expression patterns
Source: Neuroimage Clin. 2025 Nov 26;48:103916. doi: 10.1016/j.nicl.2025.103916 (PMC12720368; doi:10.1016/j.nicl.2025.103916)
Supplement: Supplementary Data 1 [file mmc1.docx]

**Distinct cortical morphometric inverse divergence changes in parkinson’s disease correlate with transcriptional expression patterns**

**Supplementary Figures**


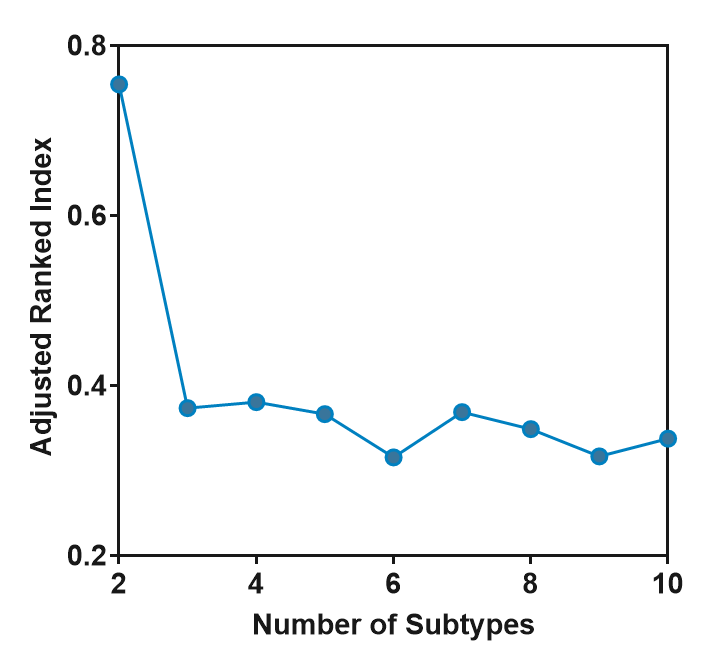


**Supplementary Figure S1.** Plots of the number of clusters versus adjusted Rand index (ARI). HYDRA determined that the two subtypes within the PD group were the optimal classification.


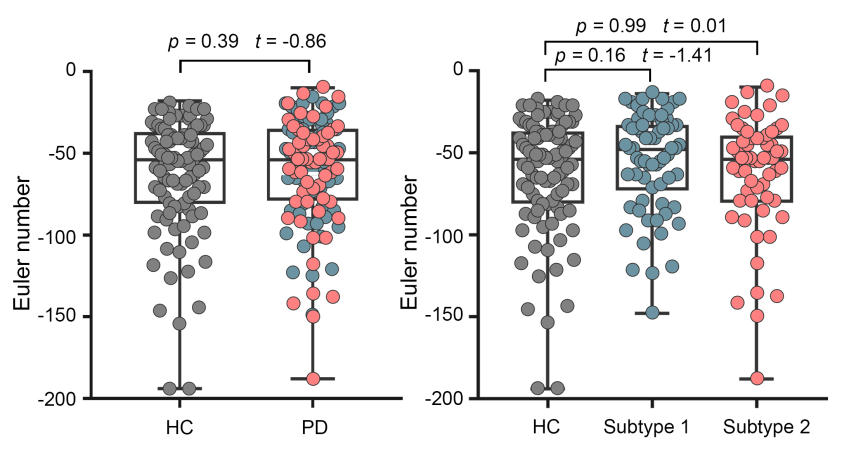


**Supplementary Figure S2.** . Euler number comparisons between PD and HC. Using a two-sample t-test, we no significant differences in the Euler number between all PD patients/ PD subtype 1/ PD subtype 2 and HC. Boxplots show the lower quartile (25th percentile), median, and upper quartile (75th percentile). The upper and lower whiskers denote the minimum and maximum, respectively.


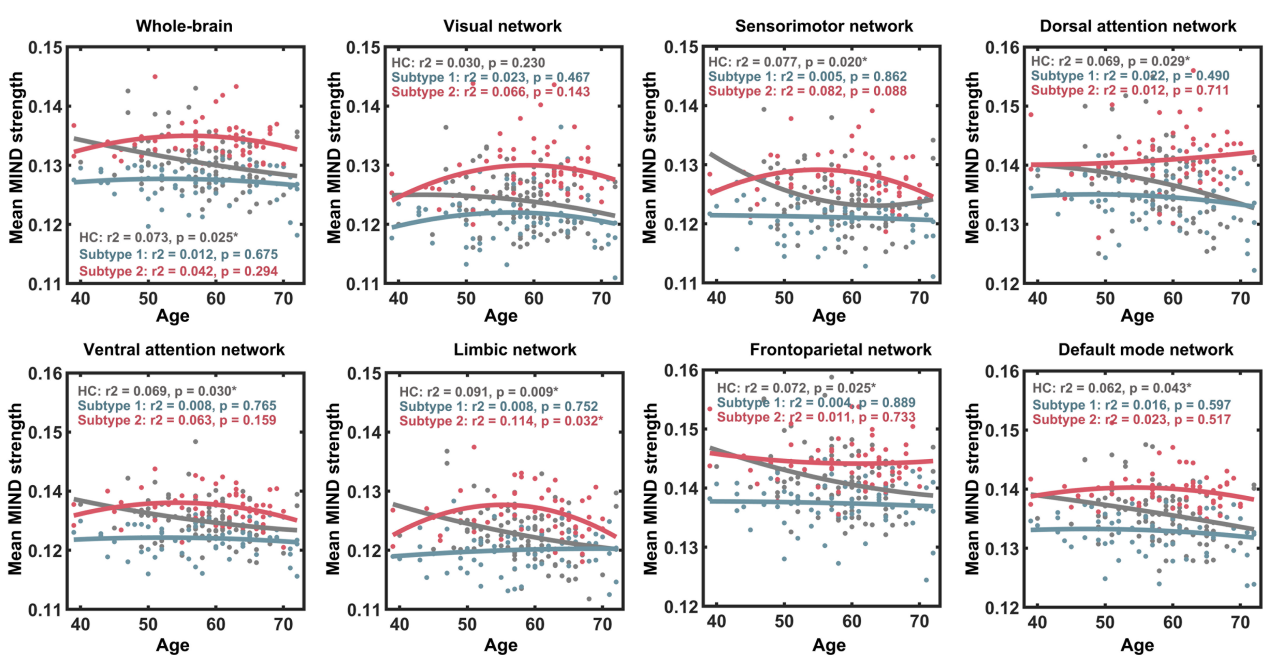


**Supplementary Figure S3.** Quadratic nonlinear model curve of the MIND strength development trajectory with age. * denoted *p* < 0.05.

**

**

**Supplementary Figure S4. A-B.** Box plots for global mean MIND score. The global MIND of PD subtype 1 exhibited a significant decreased (*t* = -5.84, *p* < 0.0001) compared to HC, while the global MIND of subtype 2 showed significant increased (*t* = 6.56, *p* < 0.0001) compared to HC.





**Supplementary Figure S5**. Yeo functional networks atlas of subtyping PD-control differences in the MIND strength (Bonferroni correction, * denoted *p* < 0.05).

**

**

**Supplementary Figure S6. The spatial correlation analysis between case-control MIND maps of PD subtypes and statistical maps of MIND strength and UPDRS**-**III, H&Y, MoCA scores.** A**.** In PD subtype 1, the case-control MIND maps exhibited a significant negative spatial correlation with UPDRS-III and H&Y scores, and a positive correlation with MoCA scores. B. In subtype 2, the case-control MIND maps exhibited no spatial negative-correlation with all clinical scores.


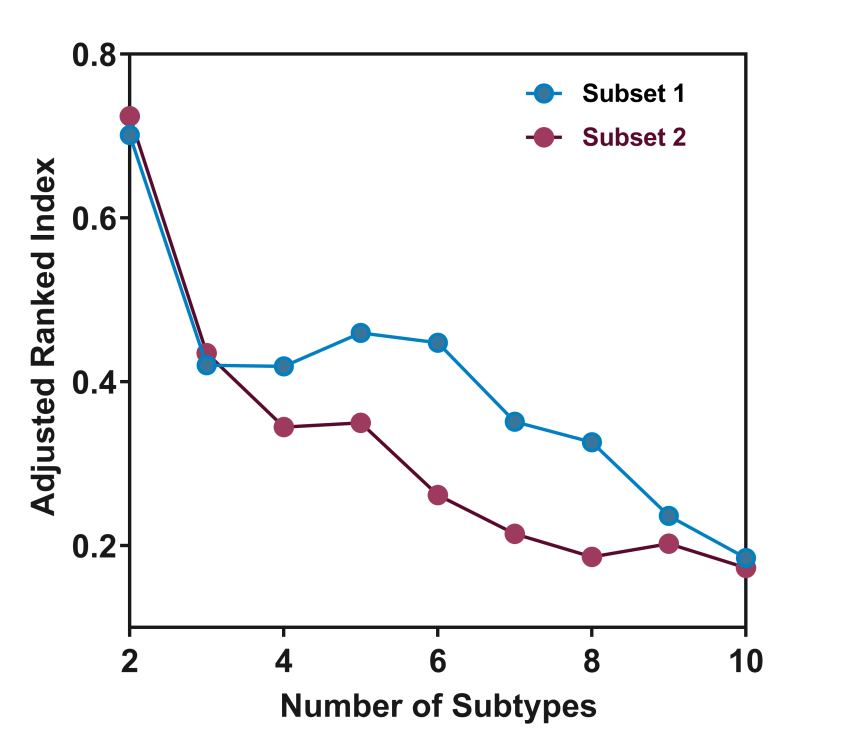


**Supplementary Figure S7.** Plots of the number of clusters versus adjusted Rand index (ARI) in Subset 1 and Subset 2. HYDRA determined that the two subtypes within the PD group were the optimal classification.





**Supplementary Figure S8.** **Comparison of MIND differences between PD subtypes in all participants and in Subsets**. (A-B) Case-control t-map of the MIND strength for all participants and subsets. (C-D) The spatial correlation analysis shows significant spatial positive-correlations of case-control t-map in both PD subtype 1/2 in all participants and in subsets.





**Supplementary Figure S9.** **Transcriptional expression patterns related to MIND strength differences in the pooled PD group.** (A) Cortical case-control t-maps and regional PLS1 gene expression weighted values in the left hemisphere for PD. Scatterplots showing the significant spatial correlation between PLS1 scores and the case-control t-maps of MIND strength in PD. (C) Spearman’s correlation analysis showed the expression of PD-related genes from AHBA datasets was positively or negatively associated with regional changes in MIND. PD showed 7 negative and 2 positive overlapping genes. All p-values were obtained from spatial correlation tests and adjusted with FDR correction. PD, Parkinson’s disease. * indicates *p* < 0.05.


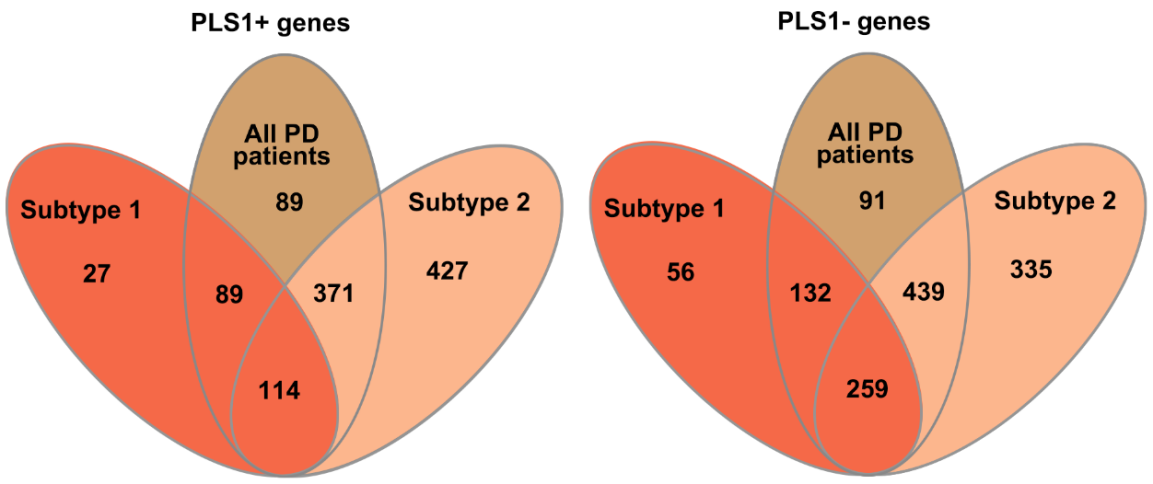


**Supplementary Figure S10.** Result displays common genes related to MIND strength differences among PD subtype 1, PD subtype 2 and all PD patients, including 114 PLS+ genes and 259 PLS- genes**.**





**Supplementary Figure S11.** **Functional enrichment of PLS1 weighted genes related to regional MIND changes in the pooled PD group.** The top 10 ontology terms for (A) PLS1+ genes (Z < -3, *p_FDR_* < 0.005) / PLS1- (B) genes (Z > 3, *p_FDR_* < 0.005). Metascape enrichment network visualization showing the same color belong to the same cluster. The size of the circle represents the number of genes involved in a given term.





**Supplementary Figure S12. Cell type-specific expression associated with MIND changes in the pooled PD group.** (A) The number of overlapping genes with PLS1+ weighted genes for each cell type in PD, including astrocytes (number = 24, adjusted *p*_perm_ = 1); endothelial cells (number = 38, adjusted *p*_perm_ = 1); microglia (number = 31, adjusted *p*_perm_ = 1); excitatory neurons (number = 71, adjusted *p*_perm_ < 0.001); inhibitory neurons (number = 58, adjusted *p*_perm_ < 0.001); oligodendrocytes (number = 22, adjusted *p*_perm_ = 1); OPCs (number = 3, adjusted *p*_perm_ = 1). (B) The number of overlapping genes with PLS- weighted genes for each cell type in PD, including astrocytes (number = 83, adjusted *p*_perm_ < 0.001);endothelial cells (number = 50, adjusted *p*_perm_ = 1); microglia (number = 35, adjusted *p*_perm_ = 1); excitatory neurons (number = 105, adjusted *p*_perm_ < 0.001); inhibitory neurons (number = 79, adjusted *p*_perm_ = < 0.001); oligodendrocytes (number = 13, adjusted *p*_perm_ = 1); OPCs (number = 9, adjusted *p*_perm_ = 0.727). (C-D) Gene ontology and pathway terms enriched for changes in MIND-related genes for the different cell types. All *p*-values were obtained from permutation tests and adjusted with FDR correction (*p* < 0.05).
